# MXene-enhanced ePatch with antibacterial activity for wound healing

**DOI:** 10.3389/fchem.2023.1280040

**Published:** 2023-10-19

**Authors:** Jing Feng, Rui Liu, Xuefeng Yuan, Changkui Cao, Ji Xie, Zhaorui Sun, Sai Ma, Shinan Nie

**Affiliations:** ^1^ Department of Emergency Medicine, Jinling Hospital, Medical School of Nanjing University, Nanjing, China; ^2^ Department of Emergency Medicine, The First School of Clinical Medicine, Southern Medical University, Nanjing, China; ^3^ Department of Cardiology, Jinling Hospital, Medical School of Nanjing University, Nanjing, China; ^4^ Department of Cardiology, The First School of Clinical Medicine, Southern Medical University, Nanjing, China

**Keywords:** MXene, ePatch, wound healing, hydrogel, enhanced

## Abstract

Prudent wound-healing strategies hold great potential in expediting tissue renovation and regeneration. Despite the widespread adoption of hydrogels as preferred carriers for wound healing patches, achieving optimal mechanical compatibility and superior wound performance remains a formidable challenge. Consequently, meticulous attention must be given to the formulation of hydrogel structure and materials design to overcome these hurdles. In response, we have developed an ePatch composed of polyacrylamide (PAAM) as the primary hydrogel structure, augmented with MXene, silver nanowires (AgNWs), and resveratrol to act as sustained-release agents, structural enhancers, and antibacterial agents, respectively. Notably, the ePatch exhibited exceptional wound-fitting capabilities and impressive mechanical stretchability (with a relative standard deviation [RSD] of only 1.36% after 55 stretches) and Young’s modulus. In contrast to the commercial 3M Tegaderm, the ePatch demonstrated superior wound healing properties, with the inclusion of MXene into PAAM/AgNWs playing a pivotal role in expanding the ePatch’s potential use across various interconnected fields.

## 1 Introduction

Wound healing is a complex biological process that involves multiple cellular and molecular mechanisms and plays a critical role in the restoration of tissue integrity and function following injury (Li et al., 2020; Zhao et al., 2020; Griffin et al., 2021). Practical wound-healing approaches can facilitate the development of new therapeutic strategies for promoting tissue repair and regeneration in various pathological conditions (Guo B. et al., 2021; Dong and Guo, 2021; Liang et al., 2021). Although there are commercially available patches (such as Mepilex and 3M Tegaderm) for wound healing, there is still room for improvement in terms of price, wound interfacial adaptability, and sustained release capability (Deng et al., 2022; Tyeb et al., 2023). Hydrogels are three-dimensional crosslinked polymer networks that absorb and retain large amounts of water or biological fluids. Due to their biocompatibility and tunable properties, hydrogels have emerged as promising biomaterials for various biomedical applications, including drug delivery, tissue engineering, and wound healing (Zhao et al., 2018; Freedman et al., 2022; Yang et al., 2022). Hydrogels can act as scaffolds to support cell growth and tissue regeneration while providing a moist environment that promotes wound healing. Additionally, hydrogels can be functionalized with bioactive molecules to enhance cellular responses and accelerate the wound-healing process (Pan et al., 2020; Xu et al., 2022). Despite their potential advantages, hydrogels face several challenges in wound healing applications, there are still several difficulties to solve: 1) how to release the active molecules slowly, 2) how to maintain the high adhesion to the skin, 3) how to optimize the wound healing properties of hydrogels. Therefore, the design of hydrogel material and the mechanical properties of its interface with skin are the essence.

Polyacrylamide (PAAM) hydrogel is a hydrophilic polymer that can absorb large amounts of water while maintaining its structural integrity (Azar et al., 2021; Ohm et al., 2021). PAAM hydrogel has the following advantages (Lim et al., 2016; Gan et al., 2018; Sun et al., 2021): a) High elasticity: PAAM has high elasticity and can withstand certain tensile and compressive stresses without breaking; b) Flexibility: PAAM has good flexibility and can adapt to wounds of different shapes and its flexibility also makes it easier to apply to uneven or curved wound surfaces (Kim et al., 2016); c) Favorable adhesion: PAAM can form good adhesion on the wound surface, which allows the gel to remain on the wound surface so that it is not easy to slip; d) Acceptable biocompatibility: PAAM has good biocompatibility and can seamlessly connect with surrounding tissues without excessive inflammation or rejection (Ohm et al., 2021). MXene is an emerging two-dimensional material that can be used to prepare hydrogels due to its unique chemical composition and structural characteristics (Wang et al., 2019; Chen et al., 2021; Zhang et al., 2022). MXene has the following advantages: 1) It has good adsorption and barrier properties, which can effectively absorb pollutants and block the penetration of harmful substances; 2) It has good biocompatibility and biodegradability and can be rapidly decomposed and decomposed in the body, which will not cause damage to the body (Lin et al., 2022); 3) It has excellent electrical conductivity and mechanical strength and can be applied in the fields of electronics and machinery, such as flexible electronics, sensors and smart materials (Guo Y. et al., 2021); 4) The surface is easy to modify and functionalize and can be used by chemical reactions to introduce various chemical functional groups on its surface, endowing it with specific properties and functions (Chen et al., 2020). Therefore, PAAM/MXene is anticipated to provide a three-dimensional hydrogel network with excellent mechanical properties and favourable biocompatibility for wound healing.

Selecting appropriate and highly effective antimicrobial agents is key to wound healing. As an emerging antibacterial material, silver nanowires (AgNWs) have the following advantages: i) Efficient antibacterial: AgNWs have an excellent antibacterial effect and can quickly kill various bacteria, viruses, fungi, and other microorganisms. It can destroy bacterial cell membranes and interfere with bacterial metabolic processes, thereby effectively inhibiting bacterial growth and reproduction (De Mori et al., 2019); ii) Safe and environmentally friendly: Compared to traditional antibacterial materials, such as antibiotics and chemical antibacterial agents, silver nanowires are more environmentally friendly and biocompatible, will not cause toxicity or allergic reactions to the human body (Azadi et al., 2020); iii) Long-lasting antibacterial: Silver nanowires can permanently inhibit bacterial growth and reproduction, and will not lose antibacterial effect over time; iv) Antibacterial properties can be adjusted: The antibacterial properties of silver nanowires can be adjusted by controlling their particle size, morphology, and surface modification to meet different application requirements (Kim et al., 2017; 2018). In summary, silver nanowires, as a new type of antibacterial material, have the advantages of high efficiency, safety, durability, wide application range, and controllability and have been widely studied and applied in various fields. However, the bactericidal effect of silver nanowires alone may not be sufficient to provide the most efficient wound healing. Resveratrol, a polyphenolic compound naturally occurring in many plants, has been shown to have a variety of biological activities, including antioxidant, anti-inflammatory, anti-cancer, and more (Duarte et al., 2015; Park et al., 2016; Vestergaard and Ingmer, 2019). In addition, resveratrol has also been found to have a specific bactericidal effect. Its mechanism of action mainly includes the following aspects: destroying cell membranes, interfering with metabolism, inhibiting enzyme activity, and inducing cell self-destruction (Duarte et al., 2015). In summary, silver nanowires exhibit potent antibacterial properties because silver ions (Ag^+^) can disrupt bacterial growth and replication. Silver ions can interact with the cell membranes and intracellular molecules of microorganisms, thereby leading to cell death. MXene can facilitate ion release and generate antibacterial effects, while PAAM itself typically lacks significant antibacterial activity but can serve as a substrate or carrier for anchoring other antibacterial agents.

In this work, we reported an MXene-assisted resveratrol-based AgNWs/PAAM hydrogel patch for advanced wound healing. In terms of materials, PAAM and MXene materials are the architectural materials of the hydrogel patch, and AgNWs and resveratrol provide excellent antibacterial properties. Moreover, the accordion-like morphology of MXene can function similarly to “clay” within the three-dimensional structure of the hydrogel, thereby enhancing its mechanical properties. In terms of skin adhesion, the hydrophilicity of polyacrylamide ensures good contact of the hydrogel with the skin and remains stable during skin stretching, while MXene and AgNWs also assist with mechanical properties. Regarding antibacterial properties, the hydrogel patch showed superior antibacterial and wound healing performance than commercial patches. Therefore, our AgNWs/PAAM hydrogel is expected to provide a novel MXene-based strategy for wound healing.

## 2 Materials and methods

### 2.1 Synthesis of AgNWs

The AgNWs were prepared as described in the previous documents (Kim et al., 2013; Jiu et al., 2014). First, 50 mL of, EG (ethylene glycol, https://www.sinoreagent.com, Shanghai, China, purity ≥99.5%) was mixed with 0.99 g PVP (polyvinyl pyrrolidone, https://www.sinoreagent.com, Shanghai, China, Guaranteed reagent) and then heated and heat-stable at 180°C. At this temperature, 0.05 g nitric acid (HNO_3_, https://www.sinoreagent.com, Shanghai, China, purity ≥65.0 ∼ 68.0%) is added, and magnetic agitation is carried out in the flask to initiate the nucleation of the silver seed. Then, adding 1.35 g of AgNO_3_ (https://www.aladdin-e.com/, Shanghai, China, purity ≥99.8%) for 80 min ensures the completion of nanoparticle growth. The obtained solution is then centrifuged three times at 3,600 revolutions per minute with alcohol.

### 2.2 Synthesis of 2D MXene

As per the methodology outlined in previously published research papers (Wang et al., 2015; Chen et al., 2018; Xu et al., 2021), we prepared Mxene from Ti_3_AlC_2_ via hydrofluoric acid (HF) etching. The process involved adding 1.5 g of Ti_3_AlC_2_ to 0.1 M HF. The mixture was then rinsed 14 times with deionized water within a temperature range of 45°C for 20 h. The solution was agitated continuously until a pH of 6.8 was attained. The resulting black powder was collected and dried under vacuum furnace conditions for 5 h at 45°C for use in the subsequent fabrication process of MXene@AgNWs@PAAM@resveratrol hydrogel. The final material was subjected to five rounds of ultrapure water washing and stored in a refrigerator at 4°C.

### 2.3 Fabrication of MXene@AgNWs@PAAM@resveratrol hydrogel

The MXene@AgNWs@PAAM@resveratrol hydrogel was obtained using the one-pot method. In a typical manner, AgNWs/resveratrol solution was mixed with acrylamide (AM, 2.0 g), N, N′-methyl-acrylamide (MBA, 0.075 g), and ammonium persulfate (APS, 0.2 g) in an ice water bath. Lastly, tetramethylethylenediamine (TEMED 20 μL) was added for 10 min under vigorous agitation (Hoang et al., 2017; Liu et al., 2019; Pantanowitz et al., 2020). The gelation can be realized by polymerizing an AM monomer after agitation. The gel system is then closed and kept for some time at 25°C. As a result, it is possible to notice that the opaque and opaque material (coded as MXene@AgNWs@PAAM) has slowly turned black with time. Pure PAAM, AgNWs, and PAAM@resveratrol hydrogel were also produced as controls.

### 2.4 Characterization

The scanning electron microscopy (SEM) analysis for hydrogel was performed on JSM-7800F with a primary energy of 15 kV. The mechanical properties of hydrogel were characterized by i-Strentek 1,510. Leica DM6B tested the biocompatibility of hydrogel with staining of Propidium iodide and Calcein-A.

### 2.5 Cell viability and H&E staining

For the cell validation of hydrogel, the Cell suspension was prepared with a density of 1 × 10^5^ ∼ 1 × 10^6^ cells/mL. 100 uL of the dyeing solution was added into 200 uL cell suspension, mixed, and incubated at 37°C for 15 min. Dead cells were stained with a P.I. solution of 5.0 uM to obtain the optimal working concentration for staining only the nucleus, not the cytoplasm. Dead cells were stained with 3.0 uM Calcein-AM to obtain the optimal working concentration without cytoplasmic staining. This concentration is then used to stain living cells. Live cells (yellow-green fluorescence) and dead cells (red fluorescence) were detected by 490 ± 10 nm excited filters under fluorescence microscopy. In addition, only dead cells were observed using 545 nm emission filters.

For H&E staining (Lai et al., 2018), first, the paraffin sections of the sample were dewaxed to water, then the nucleus was stained with hematoxylin, then the sections were dyed with Harris hematoxylin for 5 min, then the cytoplasm was stained with eosin, then the sections were stained with eosin dye solution for 2 min, and finally the sections were dehydrated and sealed. The samples were examined by microscope, and the images were collected and analyzed.

### 2.6 Animal model of wound infection and antibacterial activity

A suspension of *E. coli* (*Escherichia coli*) was prepared at an initial density of 5 × 10^8^ colony-forming units (CFU) per millilitre. Twelve C57BL/6 rats were procured and prepped over the course of 1 week. Following anaesthesia with 2.2% isoflurane and 100% O_2_, a full-thickness circular wound with a diameter of 6.8 mm was created on the back of each rat. The bacterial suspension was then sprayed onto the wound surface at a volume of 100 uL. Each group comprised three rats. The antimicrobial activity of *E. coli*, identified as ATCC BAA-35218, was examined both *in vitro* and *in vivo* (Liu et al., 2021; Wang et al., 2022). Bacterial cultures were conducted under standard temperature conditions, and the efficacy of the antimicrobial activity was assessed by comparing bacterial surface areas.

### 2.7 Statistical analysis

The data analysis was carried out using GraphPad Prism (United States). Mean ± standard deviation (S.D.) values were computed from three independent experiments (Stefan-van Staden et al., 2022). The statistical significance was evaluated by t-testing with employed. *p < 0.05, **p < 0.01, and ***p < 0.001.

## 3 Results and discussion

### 3.1 Synthesis of AgNWs, MXene, and MXene@AgNWs@PAAM@resveratrol hydrogel

**FIGURE 1:**
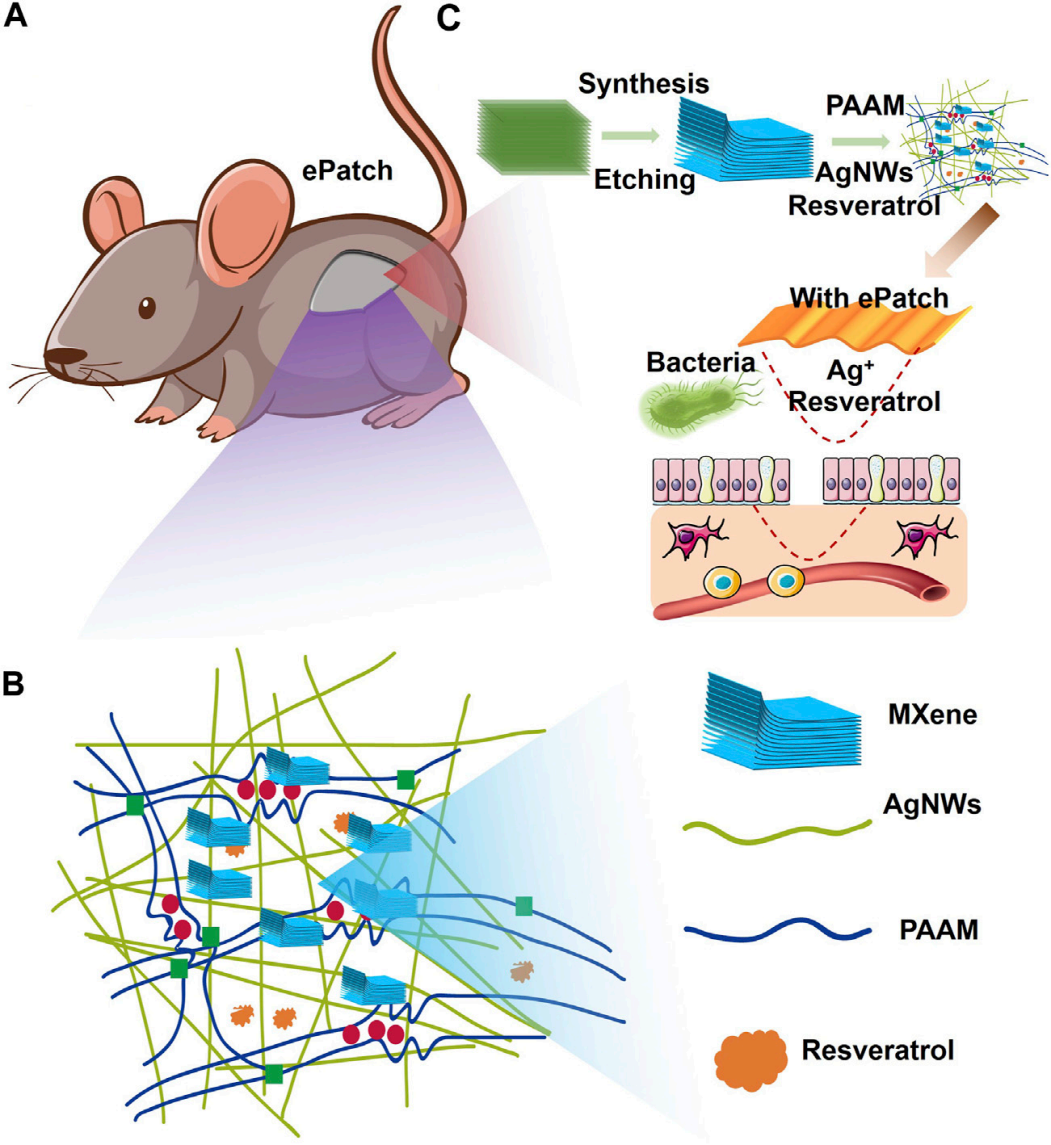
Schematic illustration of MXene-assisted AgNWs/PAAM hydrogel patch for advanced wound healing. **(A)** ePatch was attached to the mouse wound, **(B)** the component structure of ePatch including the Mxene, AgNWs, PAAM, and resveratrol. **(C)** The MXene-assisted AgNWs/PAAM hydrogel patch synthesis and its workflow.

**FIGURE 2:**
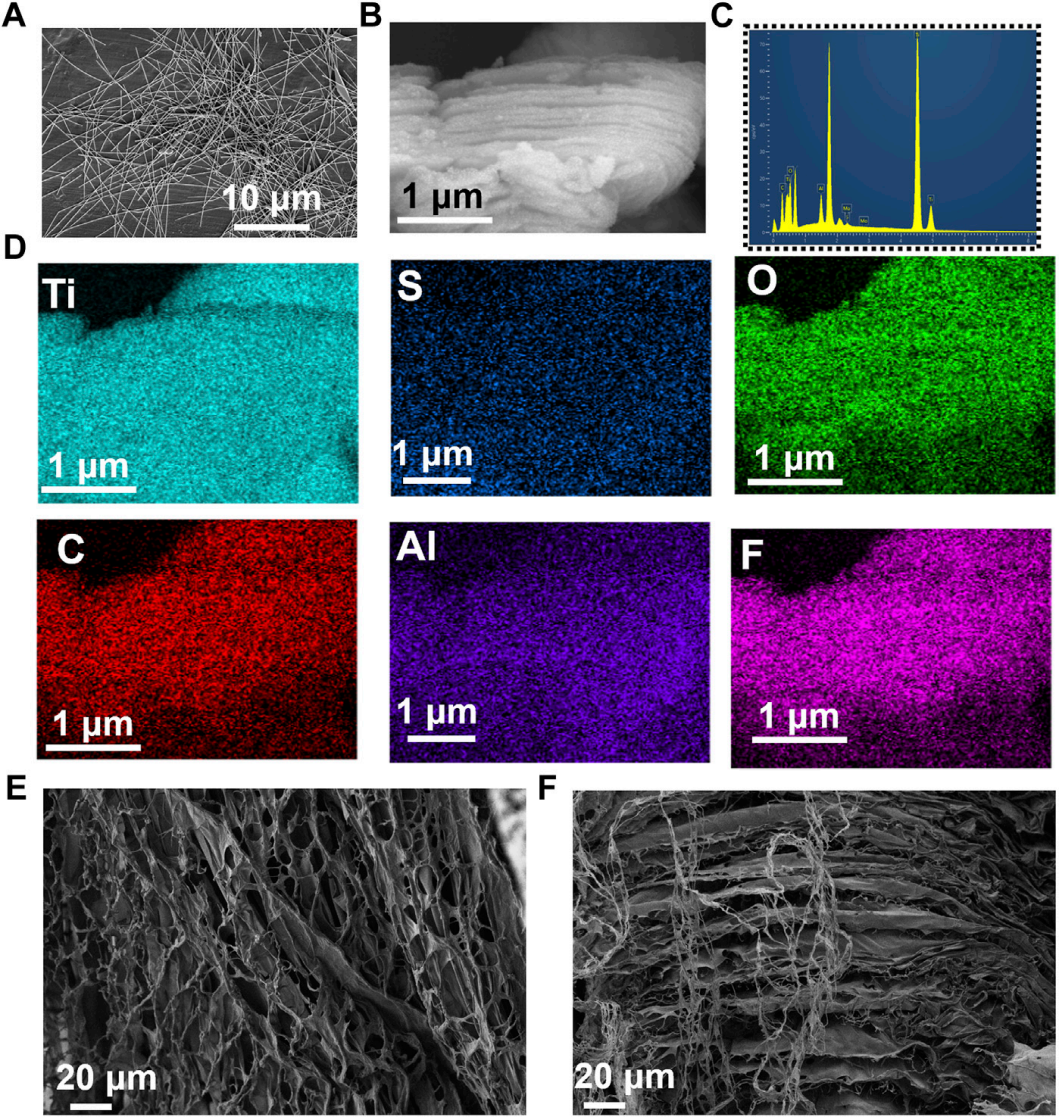
Characterizations of ePatch materials. **(A)** SEM photograph of AgNWs with an average diameter and a length of 24.3 ± 4.5 μm and 105.4 ± 11.2 nm. **(B)** SEM image of two-dimensional MXene, **(C)** elemental distribution, and **(D)** elemental mapping analysis of MXene, including the Ti, S, O, C, Al, F. **(E)** Top view and **(F)** side view images of MXene@AgNWs@PAAM@resveratrol hydrogel for advanced wound healing.

To build an efficient wound healing policy, we developed MXene@AgNWs@PAAM@resveratrol hydrogels as illustrated in Figure 1. The resulting patch is tightly attached to the wound area (Figure 1A), and the tight-fitting effect is the premise and foundation of wound healing. The active components of the hydrogels are silver nanowire, resveratrol, MXene, and resveratrol. (Figure 1B), which ultimately contribute to the enhancement of the hydrogels’ bactericidal properties, as demonstrated in Figure 1C. Silver nanowires and resveratrol in hydrogels can be used as germicidal molecules. The straight structure of the nanowire can help to enhance the performance of the hydrogels, as evidenced in Figure 1B. Thus, as an additive for hydrogels, silver nanowires have been prepared with an average diameter and length of 24.3 ± 4.5 μm and 105.4 ± 11.2 nm as illustrated in Figure 2A. In the MXene@AgNWs@PAAM@resveratrol hydrogels, the silver nanowires showed no significant changes even after 4 months of storage (Supplementary Figure S1A). As a slow-release vehicle for resveratrol, MXene increases its antimicrobial activity (Figure 1C). The morphology of the synthesized MXene was examined and is depicted in Figure 2B. The multilayer structure of MXene and the antibacterial and linear structure of AgNWs will contribute to the wound healing functionality of the patch.

We further analyzed the composition and mapping of the MXene components, which included Ti, S, O, C, Al, and F, as illustrated in Figures 2C, D. Elemental mapping and ratio analysis verified the multilayer structure of MXene, which facilitated the slow release of drugs. We utilized APS to polymerize acrylamide and to synthesize the composite as an additive to the matrix, as depicted in Figures 2E, F; Supplementary Figures S1B, S2. The morphology of the upper and lateral profiles of MXene@AgNWs@PAAM@resveratrol hydrogels was investigated, and the results indicate that the multilayered structure of MXene@AgNWs@PAAM@resveratrol is advantageous for promoting wound healing.

### 3.2 Characterization of MXene@AgNWs@PAAM@resveratrol hydrogel

**FIGURE 3:**
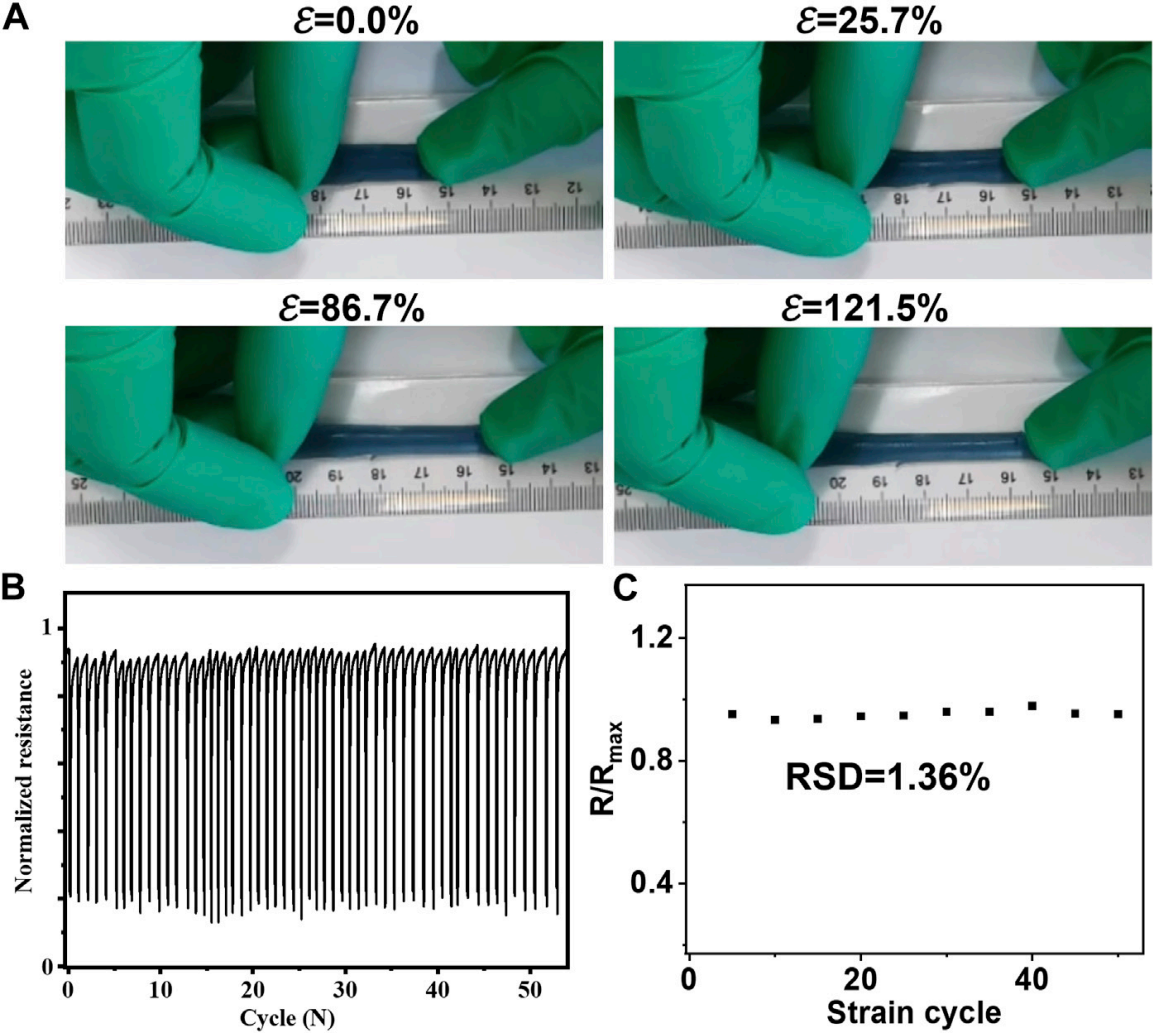
Mechanical characterizations for ePatch. **(A)** Favorable stretchable ability of MXene@AgNWs@PAAM@resveratrol to accommodate the skin’s erratic stretching with an extending of 121.5%. **(B)** There was no significant change in the resistance of the MXene@AgNWs@PAAM@resveratrol under 55 stretchings & resilience and found that the **(C)** relative standard deviation (RSD = 1.36%) is still quite low, indicating that the basic characteristics of the hydrogel are essentially unchanged in the case of continuous stretching and relaxation.

Given the continuous contraction and relaxation of skin, it is imperative for patches intended for wound healing to meet the following stringent criteria: 1) biocompatibility with skin tissue; 2) retention of properties under constant tension; 3) Young’s modulus matching that of the skin; and 4) reasonable interfacial energy for adherence to the biological interface. Notably, MXene@AgNWs@PAAM@resveratrol hydrogels exhibited highly favorable adhesive properties and can elongate up to 121.5% (as demonstrated in Figure 3A). Moreover, the hydrogels exhibited stable resistance even after 55 cycles of stretching and relaxation (as illustrated in Figure 3B), with a relatively low relative standard deviation (RSD = 1.36%) that attests to the hydrogel’s consistent properties during prolonged usage (as indicated in Figure 3C).

**FIGURE 4:**
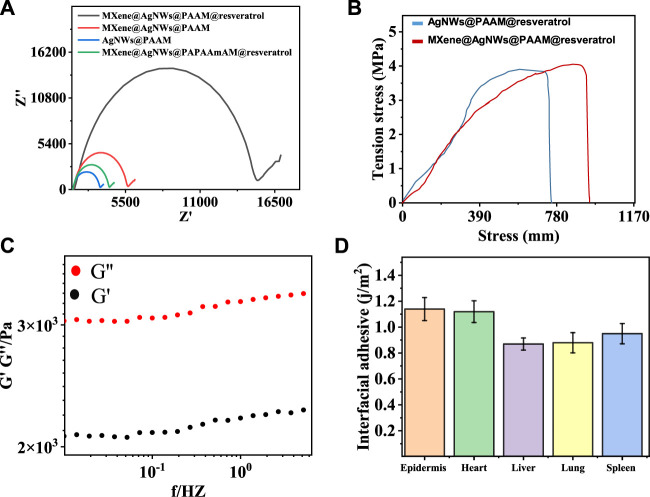
EIS, modulus and adhesion characterizations for ePatch. **(A)** Electrochemical impedance spectrometry (EIS) analysis for step-by-step fabrication of ePatch. **(B)** Young’s modulus characterization of ePacth with a value of 71.2 kPa, compatible with the skin modulus. **(C)** Viscous and elastic modulus analysis of ePatch demonstrated that the viscosity dominates properties. **(D)** The interfacial adhesive energies for different bio-interfaces were measured, including the epidermis, heart, liver, lung, and spleen, demonstrating favourable adhesive properties of ePatch.

To characterize MXene@AgNWs@PAAM@resveratrol hydrogel, we conducted electrochemical impedance spectrometry (EIS) as shown in Figure 4A. Pristine PAAm hydrogel displayed high resistance, whereas the presence of AgNWs and MXene resulted in reduced resistance, likely due to their ability to facilitate electron transfer. Conversely, resveratrol inhibits electron transfer and increases resistance. The mechanical properties of the hydrogel are comparable to those reported in other studies. We also evaluated Young’s modulus of MXene@AgNWs@PAAM@resveratrol hydrogel using the strain-stress curve (Figure 4B), and the value of 0.142 MPa was obtained using Eq. 1, which is compatible with the modulus of the skin (Sim et al., 2020; Kim et al., 2021).

$$E=\frac{\sigma }{\varepsilon }=\frac{\text{stress}}{\text{strain}}=\frac{F/A}{dl/l}$$ (1)

Young’s modulus (E) characterizes the ability of a material to deform and stretch under external forces and is calculated as the ratio of tensile stress (σ) to tensile strain (ε). Stress is defined as the amount of force applied per unit area (σ = F/A), while a strain is a change in length per unit of initial length (ε = Δl/l).

Furthermore, the viscous modulus G” of the hydrogel exceeded the elastic modulus G’, indicating that the viscosity dominates its properties (Figure 4C). Simultaneously, the intimate attachment of the ePatch to the bio-interface relies on interfacial energy. We measured the interfacial adhesive energy of the ePatch to various bio-interfaces, including the epidermis, heart, liver, lung, and spleen, demonstrating its favorable adhesive property (Figure 4D). The aforementioned characterization showcased the favorable mechanical properties of MXene@AgNWs@PAAM hydrogel as an ePatch for wound healing.

### 3.3 Wound-healing evaluation of ePatch

**FIGURE 5:**
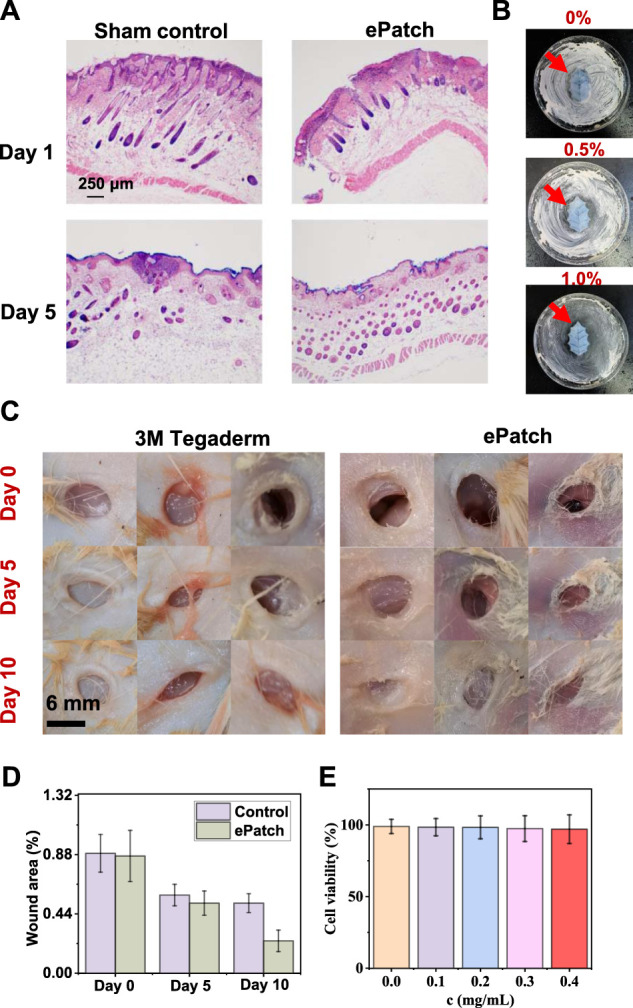
Wound-healing evaluation of ePatch. **(A)** HE staining analysis of ePatch group and control group wounds on Day 1 and Day 5. **(B)** Comparison of the antibacterial effect of ePatch with different resveratrol concentrations, including 0.0%, 0.5%, and 1.0%. **(C)** Wound healing images of the ePatch group and the commercial 3M Tegaderm group at 0, 5, and 10 days and corresponding wound area analysis. **(C,D)** After 5 days and 10 days; the results showed that the wound healing areas of the epatch after 5 days and 10 days were 0.52 ± 0.09, and 0.24 ± 0.08, respectively, better than that of 3M Tegaderm’s 0.58 ± 0.08, and 0.51 ± 0.07. **(E)** The biocompatibility analysis of MXene@AgNWs@PAAM@resveratrol hydrogel. With the increase in hydrogel concentration, the cell survival rate did not significantly decrease, demonstrating favourable biocompatibility of MXene@AgNWs@PAAM@resveratrol hydrogel.

To assess the efficacy of the MXene@AgNWs@PAAM@resveratrol hydrogel patch as an ePatch, we established an animal model of wound infection in mice. We evaluated its effectiveness through wound healing effects, Hematoxylin-Eosin (HE), and other characterizations. HE staining is capable of differentiating the nucleus and cytoplasmic components of cells, leading to the presentation of different cell types with distinct morphology and colouration under a microscope. As a result, HE staining has been widely employed to analyze the healing process of wounds. This method effectively discriminates different types of cells, including immune and inflammatory cells. As depicted in Figure 5A; Supplementary Figure S3, HE staining images from the control and experimental groups demonstrated that the ePatch group had a lower proportion of immune cells and a better healing effect at the wound site. Concurrently, we compared the inhibitory effect of different concentrations of AgNWs@resveratrol on *E. coli* and found that 1.0% AgNWs@resveratrol (Supplementary Figure S4) exhibited the best inhibitory effect compared to the control group (Figure 5B). Fine-tuning the concentration of AgNWs@resveratrol to optimal levels can significantly enhance the performance of ePatch. However, it is imperative to avoid exceeding a certain threshold to prevent any detrimental effects on other essential functions of the ePatch.

For the evaluation of the wound healing effect of animal models, we compared ePatch with commercial 3M Tegaderm, with the initial state as the selected time point (Figure 5C). The wound area serves as a direct indicator of the progress of wound healing and thereby serves as an accurate reflection of the efficacy of ePatch in promoting wound healing. After 5 and 10 days, the results demonstrated that the wound healing area of the ePatch was 0.52 ± 0.09 and 0.24 ± 0.08, respectively, which was better than that of 3M Tegaderm’s 0.58 ± 0.08 and 0.51 ± 0.07. The above qualitative and quantitative analyses demonstrated that the ePatch had a better wound-healing function than commercial 3M Tegaderm (Figure 5D). Additionally, we incubated different concentrations of ePatch patch materials and epidermal cells (Figure 5E; Supplementary Figure S5). The cell survival rates maintained a high level (>97.1%), indicating that the ePatch has good biocompatibility. The H&E mentioned above staining, concentration optimization, wound healing evaluation, and biocompatibility analysis collectively provided robust evidence that attests to the practicality of ePatch as an effective wound healing therapy.

## 4 Conclusion

In summary, we introduce an innovative approach for advanced wound healing, using an MXene-assisted resveratrol-based AgNWs/PAAM hydrogel patch. The novelty of our strategy stems from the optimized structural design and composition of the ePatch, which is tailored to promote optimal wound healing outcomes. The active components of the patch, AgNWs and resveratrol, are used as antibacterial agents, while the 3D structure of PAAM, the 2D structure of MXene, and the 1-D structure of AgNWs combine to imbue the patch with superior mechanical properties, such as adhesion, tensile strength, and Young’s modulus. Furthermore, the MXene@AgNWs@PAAM@resveratrol patch demonstrates exceptional mechanical stability, retaining its properties even after undergoing 55 repetitive stretching cycles, with a low relative standard deviation (RSD) of only 1.36%. Importantly, in animal models, our patch has shown to outperform the commercial 3M Tegaderm, thereby underscoring the tremendous potential of the MXene@AgNWs@PAAM@resveratrol strategy for future wound healing applications and the development of other MXene-based biomedical technologies.

## Data Availability

The original contributions presented in the study are included in the article/Supplementary Material, further inquiries can be directed to the corresponding authors.
