# Supplementary material for: MXene-enhanced ePatch with antibacterial activity for wound healing
Source: Front Chem. 2023 Oct 19;11:1280040. doi: 10.3389/fchem.2023.1280040 (PMC10620505; doi:10.3389/fchem.2023.1280040)
Supplement: Supplementary file 1 [file DataSheet1.PDF]

Supplementary Material

MXene-enhanced ePatch with antibacterial activity for wound healing

Jing Feng[1,2], Rui Liu[1,2], Xuefeng Yuan[1,2], Changkui Cao[1,2], Ji Xie[1,2], Zhaorui Sun[1,2], Sai Ma[3, 4*], Shinan Nie[1,2*]

[1]Department of Emergency Medicine, Jinling Hospital, Medical School of Nanjing University, Nanjing, 210002, PR China

[2]Department of Emergency Medicine, the First School of Clinical Medicine, Southern Medical University, Nanjing, 210002, PR China

[3]Department of Cardiology, Jinling Hospital, Medical School of Nanjing University, Nanjing, 210002, PR China

[4]Department of Cardiology, the First School of Clinical Medicine, Southern Medical University, Nanjing, 210002, PR China

*** Correspondence: saimamacey@163.com; shn_nie@sina.com;**

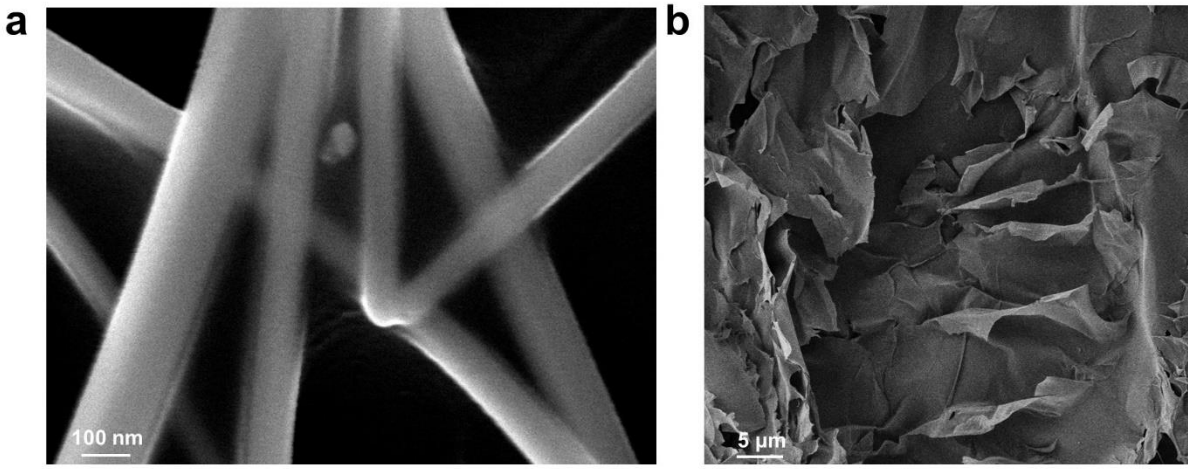

S1. a) SEM image of the AgNWs in MXene@AgNWs@PAAM@resveratrol hydrogels after four months of storage, b) Enlarged SEM image of MXene@AgNWs@PAAM@resveratrol hydrogel.

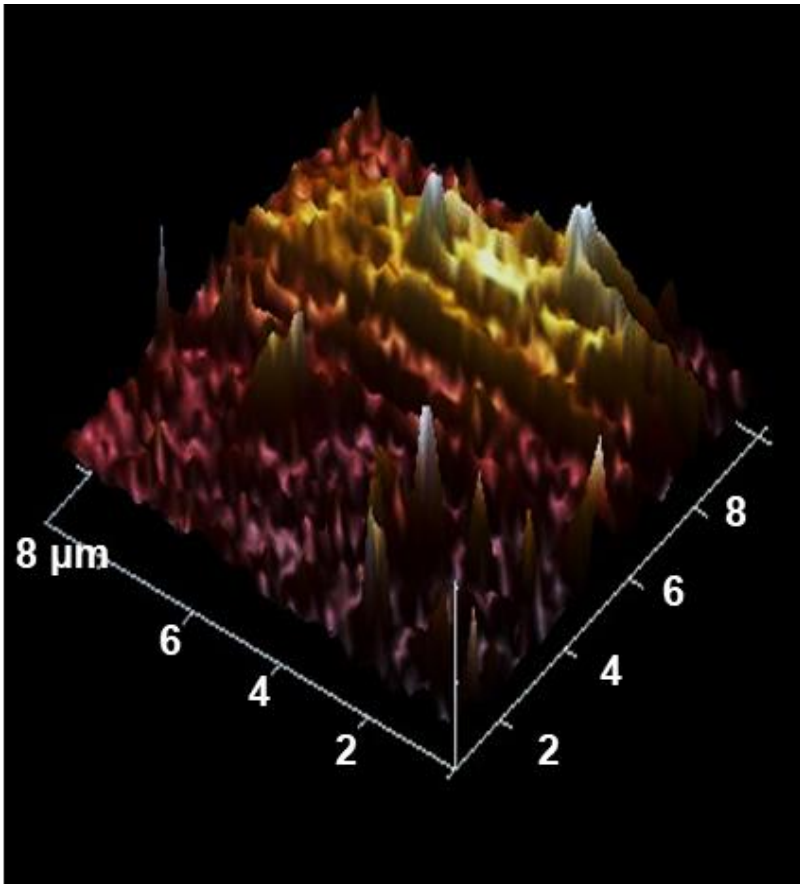

S2. AFM analysis of the MXene@AgNWs@PAAM@resveratrol hydrogel.

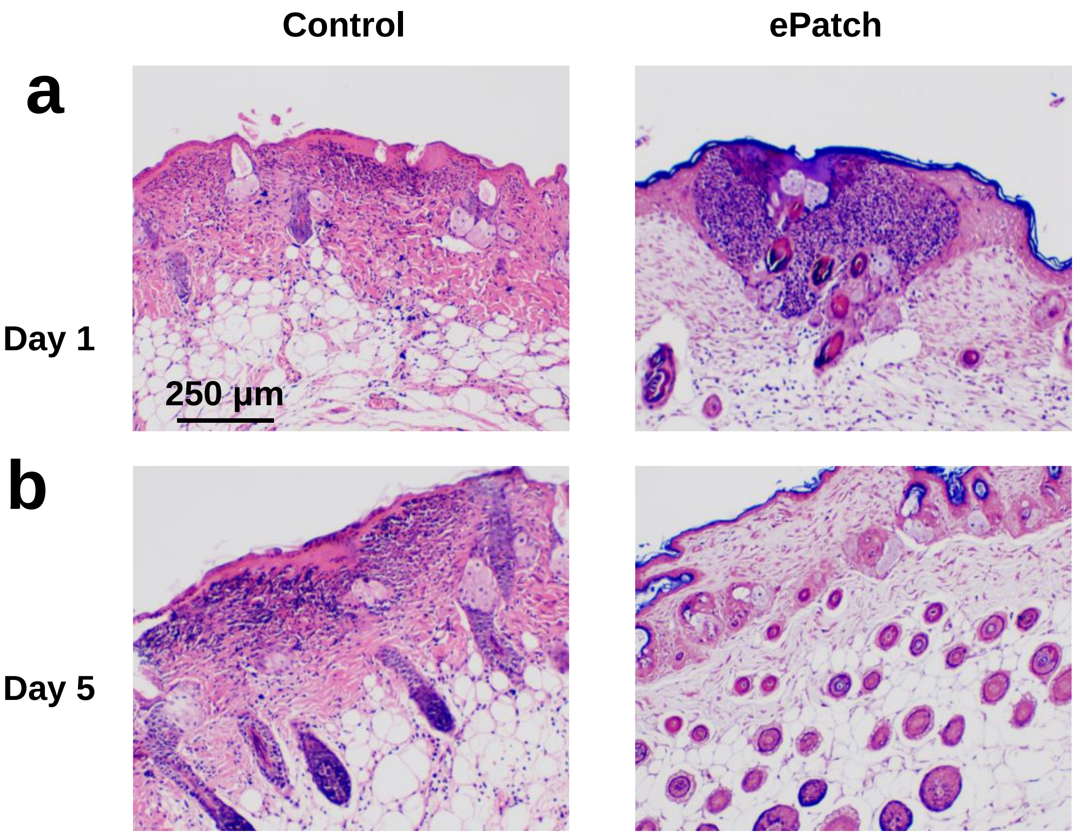

S3. The enlarged H&E staining of the wound sections in the sham control and ePatch group after a) 1 and b) 5 days.

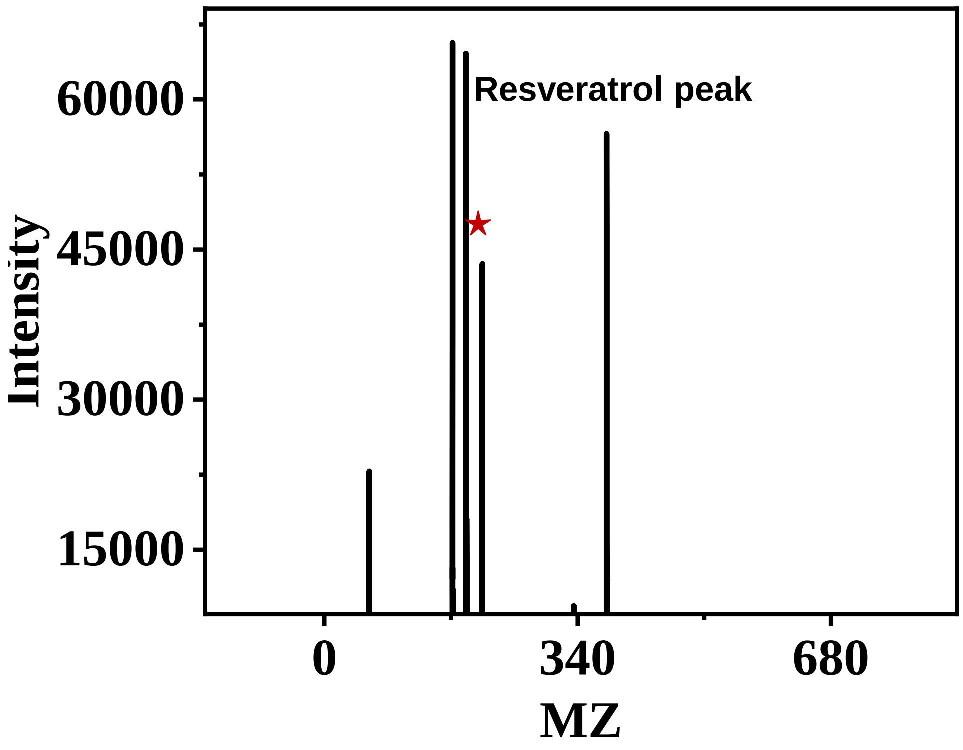

S4. Mass spectrometry analysis of the resveratrol.

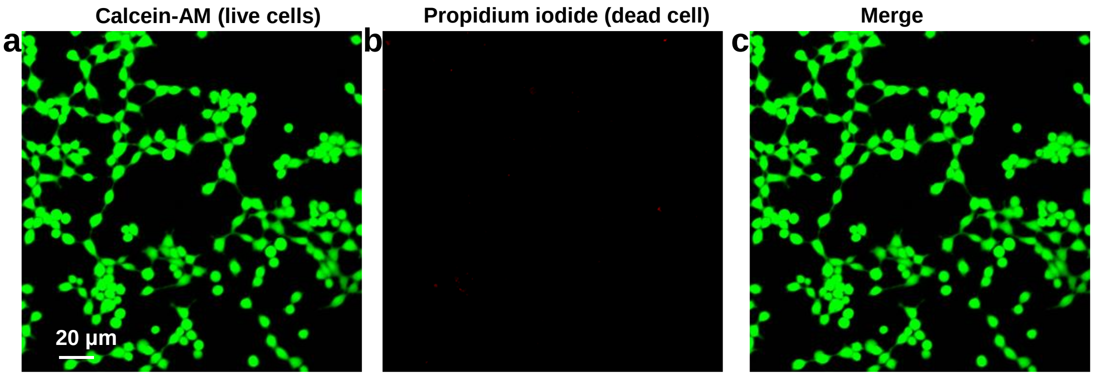

S5. The cell viability analysis for MXene@AgNWs@PAAM@resveratrol hydrogel. Photographs of a) live cells, b) dead cells, and c) merged.

Table S1. EDS analysis of MXene@AgNWs@PAAM@resveratrol hydrogel.

Element	Type	Concentration	K value	Wt%	Wt% Sigma	Atomic percentage
C	K	9.19	0.09187	12.2	0.15	24.23
O	K	5.76	0.05049	17.39	0.18	25.92
F	K	7.86	0.06113	18.24	0.12	22.91
Al	K	3.72	0.03403	2.73	0.11	2.27
S	K	0.3	0.00315	0.19	0.03	0.14
Ti	K	81.71	0.81714	49.25	0.16	24.53
Mo	K	0.35	0.0035	0.23	0.09	0.06
Total:				100	100	100

Table S1. Comparisons with other antibacterial materials.

Number	Functional materials	Functions	Reference
**1**	Antibacterial drugs	Amoxicillin	[1]
**2**	Antibacterial drugs	Ampicillin	[2]
**3**	Photothermal antibacterial	CNTs	[3]
**4**	Inorganic metals	Zinc	[4]
**5**	Photothermal antibacterial	PDA	[5]
**6**	Photodynamic antibacterial	MoS2	[6]
**7**	Photodynamic antibacterial	g-C_3N_4@AuNPs	[7]
**8**	Photodynamic antibacterial	Fe3O4@MoS2-Ag	[6]
**9**	Cationic polymer	CS/gelatin	[6]
**10**	Two-dimensional materials	MXene	This work

Reference

[1] J. Qu, X. Zhao, Y. Liang, Y. Xu, P. X. Ma, B. Guo, *Chem. Eng. J.* **2019**, *362*, 548.

[2] M. S. Refat, K. M. Elsabawy, A. Alhadhrami, A. S. A. Almalki, M. Y. El-Sayed, R. F. Hassan, *J. Mol. Liq.* **2018**, *255*, 462.

[3] Y. Liang, X. Zhao, T. Hu, Y. Han, B. Guo, *J. Colloid Interface Sci.* **2019**, *556*, 514.

[4] Y. Li, Y. Han, X. Wang, J. Peng, Y. Xu, J. Chang, *ACS Appl. Mater. Interfaces* **2017**, *9*, 16054.

[5] G. Gao, Y. W. Jiang, H. R. Jia, F. G. Wu, *Biomaterials* **2019**, *188*, 83.

[6] Y. Liang, J. He, B. Guo, *ACS Nano* **2021**, *15*, 12687.

[7] F. Wei, X. Cui, Z. Wang, C. Dong, J. Li, X. Han, *Chem. Eng. J.* **2021**, *408*, DOI 10.1016/j.cej.2020.127240.
